# Supplementary material for: A Novel and Simplified Score for Determining Treatment Eligibility for Patients with Chronic Hepatitis B
Source: Viruses. 2023 Mar 10;15(3):724. doi: 10.3390/v15030724 (PMC10056199; doi:10.3390/v15030724)
Supplement: Supplementary file 1 [file viruses-15-00724-s001.zip › viruses-2242226-supplementary.pdf]

SUPPLEMENTARY MATERIALS

Table S1 Summary of international and THASL guideline recommendations for antiviral treatment for chronic HBV infections.

Viral replication		Liver Inflammation		Liver Fibrosis	Decision
HBeAg Status	HBV DNA (IU/ml)	ALT	Histopathology	Histology or liver stiffness	
**Thai Guideline 2015**					
Positive or negative	>2,000	>2X ULN for 3 mo			Treat
			≥A2		
				≥F2	
	Detectable			F4	
**EASL 2017**					
Positive	>20,000	>2X ULN			Treat
Negative	>2,000		≥A2		
				≥F2	
Positive or negative	Detectable			F4	
**AASLD 2016**					
Positive	>20,000	>2X ULN			Treat
				≥F2	
Negative	>2,000	>2X ULN			
				≥F2	
Positive or negative	Detectable			Compensated F4	
				Decompensated F4	
				≥F2	
**APASL 2016**					
Positive	>20,000	>2X ULN for 1 mo			Treat
			≥A2		
				≥F2	
Negative	>2,000	>2X ULN for 1 mo			
			≥A2		
				≥F2	
Positive or negative	>2,000			Compensated F4	
	Detectable			Decompensated F4	

**UNL of ALT: APASL, EASL = 40, AASLD = 35 (male), 25 (female)

AASLD, American Association for the Study of Liver Diseases; ALT, alanine aminotransferase; APASL, Asian-Pacific clinical practice guidelines on the management of hepatitis; EASL, European Association for the Study of the Liver; F, fibrosis; HBeAg, hepatitis B e antigen; TB, total bilirubin; THASL, Thai Association for the Study of the Liver; ULN; upper limit normal.

Table S2. Performance of HePAA scores and optimal cutoff in derivation cohort (n = 602)

Cutoff	1	2	3	4	5	6
Sensitivity (%)	96.7	92.1	84.9	50.0	16.4	1.3
Specificity (%)	59.0	83.6	92.6	97.2	99.7	100

Table S3. Performance of simplified score for chronic hepatitis B treatment eligibility according to AASLD guideline in derivation cohort (n = 602)

	**TREAT-B (≥2)**	**Simplified WHO criteria**	**REACH-B (≥6)**	**HePAA** (≥2)	**HePAA** (≥3)
**AUROC**	0.927 (0.904-0.951)	0.781 (0.750-0.812)	0.856 (0.821-0.890)	0.933 (0.907-0.958)	
p* value *	0.545	<0.001	<0.001	N/A	
**Sensitivity (%)**	85.9 (79.7-90.7)	94.7 (90.2-97.6)	97.6 (94.1-99.4)	91.6 (86.1-95.5)	85.5 (77.8-89.8)
**Specificity (%)**	87.3 (83.8-90.3)	62.7 (58.0-67.3)	37.7 (33.1-42.5)	84.1 (79.7-87.9)	93.2 (89.8-95.6)
**Positive predictive value (%)**	72.6 (65.9-78.7)	50.0 (44.4-55.6)	38.2 (33.6-42.9)	73.6 (63.3-78.3)	85.6 (79.9-90.0)
**Negative predictive value (%)**	94.0 (91.2-96.1)	96.8 (94.0-98.5)	97.6 (94.0-99.3)	95.4 (92.5-97.2)	92.6 (89.6-94.7)
**Positive likelihood ratio**	6.75 (5.23-9.70)	2.54 (2.24-2.89)	1.57 (1.45-1.69)	5.77 (4.46-7.45)	12.33 (8.19-18.56)
**Negative likelihood ratio**	0.16 (0.11-0.23)	0.08 (0.04-0.16)	0.06 (0.02-0.17)	0.10 (0.06-0.17)	0.17 (0.11-0.24)

AUROC, area under receiver operating characteristic curves; REACH-B, risk estimation for hepatocellular carcinoma in chronic hepatitis B; TREAT-B, treatment eligibility in Africa for the hepatitis B virus; WHO, World Health Organization.
** *p* values were obtained using Stata's "roccomp" command.

Table S4. Performance of simplified score for chronic hepatitis B treatment eligibility according to AASLD guideline in validation cohort (n = 420)

	**TREAT-B (≥2)**	**Simplified WHO criteria**	**REACH-B (≥6)**	**HePAA** (≥2)	**HePAA** (≥3)
**AUROC**	0.877 (0.839-0.915)	0.705 (0.671-0.739)	0.831 (0.788-0.873)	0.885 (0.851-0.919)	
p* value*	0.553	<0.001	<0.001	N/A	
**Sensitivity (%)**	85.1 (76.3-91.6)	95.7 (89.5-98.8)	93.6 (86.6-97.6)	97.6 (92.5-99.7)	88.2 (79.8-93.9)
**Specificity (%)**	72.4 (67.2-77.2)	54.9 (49.3-60.4)	36.2 (31.0-41.7)	57.2 (51.7-62.7)	74.1 (69.0-78.8)
**Positive predictive value (%)**	47.1 (39.4-54.9)	37.3 (34.4-40.6)	29.7 (24.6-35.3)	39.5 (36.5-42.6)	49.3 (44.4-54.3)
**Negative predictive value (%)**	94.4 (90.8-96.9)	97.9 (94.6-99.2)	95.2 (89.8-98.2)	98.9 (95.9-99.7)	95.7 (92.6-97.5)
**Positive likelihood ratio**	3.08 (2.54-3.75)	2.12 (1.87-2.41)	1.47 (1.33-1.62)	2.29 (2.01-2.60)	3.41 (2.80-4.16)
**Negative likelihood ratio**	0.21 (0.13-0.34)	0.08 (0.03-0.20)	0.18 (0.08-0.39)	0.04 (0.01-0.15)	0.16 (0.09-0.28)

AUROC, area under receiver operating characteristic curves; REACH-B, risk estimation for hepatocellular carcinoma in chronic hepatitis B; TREAT-B, treatment eligibility in Africa for the hepatitis B virus; WHO, World Health Organization.
** *p* values were obtained using Stata's "roccomp" command.

Table S5. Performance of various systems for chronic hepatitis B treatment eligibility according to APASL guideline in derivation cohort (n = 602)

	**TREAT-B (≥2)**	**Simplified WHO criteria**	**REACH-B (≥6)**	**HePAA** (≥2)	**HePAA** (≥3)
**AUROC**	0.928 (0.904-0.952)	0.776 (0.745-0.807)	0.859 (0.825-0.894)	0.933 (0.907-0.958)	
p* value*	0.650	<0.001	<0.001	N/A	
**Sensitivity (%)**	87.7 (81.6-92.3)	95.7 (91.3-98.2)	98.1 (94.7-99.6)	91.9 (86.4-95.8)	85.9 (79.3-91.1)
**Specificity (%)**	86.4 (82.9-89.5)	62.1 (57.3-66.6)	37.5 (32.9-42.2)	83.3 (78.8-87.2)	92.6 (89.2-95.2)
**Positive predictive value (%)**	70.6 (63.8-76.8)	48.4 (42.8-54.1)	36.9 (32.3-41.6)	71.8 (66.5-76.5)	84.2 (78.3-88.8)
**Negative predictive value (%)**	94.9 (92.3-96.9)	97.5 (94.9-99.0)	98.2 (94.8-99.6)	95.7 (92.9-97.5)	93.4 (90.5-95.5)
**Positive likelihood ratio**	6.46 (5.06-8.25)	2.52 (2.23-2.86)	1.57 (1.46-1.69)	5.50 (4.29-7.05)	11.56 (7.82-17.08)
**Negative likelihood ratio**	0.14 (0.09-0.22)	0.07 (0.03-0.14)	0.05 (0.02-0.15)	0.10 (0.06-0.17)	0.15 (0.10-0.23)

AUROC, area under receiver operating characteristic curves; REACH-B, risk estimation for hepatocellular carcinoma in chronic hepatitis B; TREAT-B, treatment eligibility in Africa for the hepatitis B virus; WHO, World Health Organization.
** *p* values were obtained using Stata's "roccomp" command.

Table S6. Performance of various systems for chronic hepatitis B treatment eligibility according to APASL guideline in validation cohort (n = 420)

	**TREAT-B (≥2)**	**Simplified WHO criteria**	**REACH-B (≥6)**	**HePAA** (≥2)	**HePAA** (≥3)
**AUROC**	0.877 (0.839-0.915)	0.705 (0.671-0.739)	0.831 (0.788-0.873)	0.885 (0.851-0.919)	
p* value*	0.553	<0.001	<0.001	N/A	
**Sensitivity (%)**	85.1 (76.3-91.6)	95.7 (89.5-98.8)	93.6 (86.6-97.6)	97.9 (92.5-99.7)	88.3 (79.8-93.9)
**Specificity (%)**	72.4 (67.2-77.2)	54.9 (49.3-60.4)	36.2 (31.0-41.7)	57.2 (51.7-62.7)	74.2 (69.0-78.8)
**Positive predictive value (%)**	47.1 (39.4-54.9)	37.3 (34.4-40.6)	29.7 (24.6-35.3)	39.5 (36.5-42.6)	49.3 (44.4-54.3)
**Negative predictive value (%)**	94.4 (90.8-96.9)	97.9 (94.6-99.2)	95.2 (89.8-98.2)	98.9 (95.9-99.7)	95.6 (92.6-97.5)
**Positive likelihood ratio**	3.08 (2.54-3.75)	2.12 (1.87-2.41)	1.47 (1.33-1.62)	2.29 (2.01-2.60)	3.41 (2.80-4.16)
**Negative likelihood ratio**	0.21 (0.13-0.34)	0.08 (0.03-0.20)	0.18 (0.08-0.39)	0.04 (0.01-0.15)	0.16 (0.09-0.28)

AUROC, area under receiver operating characteristic curves; REACH-B, risk estimation for hepatocellular carcinoma in chronic hepatitis B; TREAT-B, treatment eligibility in Africa for the hepatitis B virus; WHO, World Health Organization.
** *p* values were obtained using Stata's "roccomp" command.

Table S7. Performance of various systems for chronic hepatitis B treatment eligibility according to THASL guideline in derivation cohort (n = 602)

	**TREAT-B (≥2)**	**Simplified WHO criteria**	**REACH-B (≥6)**	**HePAA** (≥2)	**HePAA** (≥3)
**AUROC**	0.928 (0.904-0.951)	0.783 (0.753-0.814)	0.858 (0.824-0.892)	0.936 (0.911-0.960)	
p* value*	0.4099	<0.001	<0.001	N/A	
**Sensitivity (%)**	86.5 (80.5-91.3)	95.3 (91.0-98.0)	98.2 (95.0-99.6)	91.7 (86.3-95.5)	84.7 (78.1-90.0)
**Specificity (%)**	87.2 (83.7-90.2)	63.1 (58.4-67.7)	38.1 (33.4-42.8)	84.6 (80.2-88.4)	93.7 (90.5-96.1)
**Positive predictive value (%)**	72.9 (66.2-78.9)	50.6 (45.0-56.2)	38.6 (34.0-43.4)	74.6 (69.4-79.3)	86.9 (81.2-91.1)
**Negative predictive value (%)**	94.2 (91.5-96.3)	97.1 (94.4-98.8)	98.2 (94.8-99.6)	95.4 (92.5-97.2)	92.6 (89.6-94.7)
**Positive likelihood ratio**	6.78 (5.26-8.74)	2.58 (2.27-2.94)	1.59 (1.47-1.71)	5.97 (4.60-7.76)	13.51 (8.79-20.76)
**Negative likelihood ratio**	0.15 (0.11-0.23)	0.07 (0.04-0.15)	0.05 (0.01-0.14)	0.10 (0.06-0.16)	0.16 (0.11-0.24)

AUROC, area under receiver operating characteristic curves; REACH-B, risk estimation for hepatocellular carcinoma in chronic hepatitis B; TREAT-B, treatment eligibility in Africa for the hepatitis B virus; WHO, World Health Organization.
** *p* values were obtained using Stata's "roccomp" command.

Table S8. Performance of various systems for chronic hepatitis B treatment eligibility according to THASL guideline in validation cohort (n = 420)

	**TREAT-B (≥2)**	**Simplified WHO criteria**	**REACH-B (≥6)**	**HePAA** (≥2)	**HePAA** (≥3)
**AUROC**	0.877 (0.839-0.915)	0.705 (0.671-0.739)	0.831 (0.788-0.873)	0.885 (0.851-0.919)	
p* value*	0.553	<0.001	<0.001	N/A	
**Sensitivity (%)**	85.1 (76.3-91.6)	95.7 (89.5-98.8)	93.6 (86.6-97.6)	97.9 (92.5-99.7)	88.3 (79.8-93.9)
**Specificity (%)**	72.4 (67.2-77.2)	54.9 (49.3-60.4)	36.2 (31.0-41.7)	57.2 (51.7-62.7)	74.2 (69.0-78.8)
**Positive predictive value (%)**	47.1 (39.4-54.9)	37.3 (34.4-40.6)	29.7 (24.6-35.3)	39.5 (36.5-42.6)	49.3 (44.4-54.3)
**Negative predictive value (%)**	94.4 (90.8-96.9)	97.9 (94.6-99.2)	95.2 (89.8-98.2)	98.9 (95.9-99.7)	95.6 (92.6-97.5)
**Positive likelihood ratio**	3.08 (2.54-3.75)	2.12 (1.87-2.41)	1.47 (1.33-1.62)	2.29 (2.01-2.60)	3.41 (2.80-4.16)
**Negative likelihood ratio**	0.21 (0.13-0.34)	0.08 (0.03-0.20)	0.18 (0.08-0.39)	0.04 (0.01-0.15)	0.16 (0.09-0.28)

AUROC, area under receiver operating characteristic curves; REACH-B, risk estimation for hepatocellular carcinoma in chronic hepatitis B; TREAT-B, treatment eligibility in Africa for the hepatitis B virus; WHO, World Health Organization.
** *p* values were obtained using Stata's "roccomp" command.

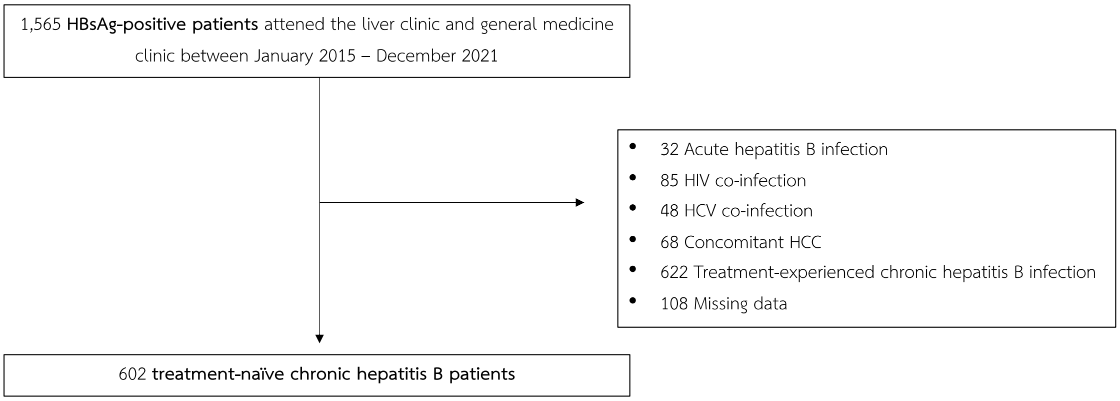

Figure S1. Study flow chart
HBV, hepatitis B virus; HCC, hepatocellular carcinoma; HCV, hepatitis C virus; HIV, human immunodeficiency virus; HBsAg, hepatitis B s antigen.
